# Prevalence of Gastrointestinal Symptoms, Hepatic Dysfunction, and Outcomes in Hospitalized Patients With COVID-19 Infection: An Early Experience

**DOI:** 10.7759/cureus.22152

**Published:** 2022-02-12

**Authors:** Krystal Hasel, Ahlaa Salim, Sandeep Verma, Christopher D'Adamo, Denise Arrup, Rakesh Vinayek, Sudhir K Dutta

**Affiliations:** 1 Internal Medicine, Sinai Hospital of Baltimore, Baltimore, USA; 2 Medicine/Gastroenterology, Sinai Hospital of Baltimore, Baltimore, USA; 3 Family and Community Medicine, University of Maryland Medical Center, Baltimore, USA; 4 Nursing, Sinai Hospital of Baltimore, Baltimore, USA; 5 Gastroenterology and Hepatology, Sinai Hospital of Baltimore, Baltimore, USA; 6 Gastroenterology and Hepatology, Sinai Hospital of Baltimore/University of Maryland School of Medicine, Baltimore, USA

**Keywords:** hepatic dysfunction, covid-19, outcomes in covid-19 hospitalizations, gi manifestations

## Abstract

Background and objective

Coronavirus disease 2019 (COVID-19) was first reported in China two years ago as primarily a lung infection associated with cough and fever. It spread rapidly across the world and was declared a pandemic in early 2020, with 131 million people infected and 2.85 million deaths worldwide. To date, approximately 550,000 deaths have occurred due to COVID-19 in the United States and the numbers continue to rise. The extrapulmonary manifestations of this disease such as acute kidney injury (AKI), cardiovascular events, and gastrointestinal (GI) indications were not emphasized initially. However, subsequent studies from the United States and Canada have noted GI involvement in this disease in a large number of cases. Our group, taking care of these patients during the early phase of the pandemic in 2020, observed the frequent presentations of GI symptoms such as diarrhea and hepatic dysfunction and this study examines the same.

Methods

We undertook a retrospective study of 184 consecutive adult patients who were hospitalized at our center with confirmed COVID-19 infection, with a view to further elucidate the GI and hepatic involvement during the early breakout (March 17-May 17, 2020) of this illness.

Results

Major comorbidities associated with this illness in our cohort of patients included hypertension (HTN, 66%), diabetes mellitus (DM, 44%), obesity (41%), and chronic kidney disease (CKD, 17%). The most common GI manifestation was diarrhea (25%) and, interestingly, more than two-thirds of the patients had at least one liver function abnormality. The most common liver function abnormality was elevated serum aspartate aminotransferase (AST). Elevated AST was significantly correlated (p<0.05) with inflammatory markers such as D-dimer, lactate dehydrogenase (LDH), and ferritin, as well as AKI by bi-variate analysis. Salient observations from our study include higher mortality, frequent AKI, and cardiovascular events in male patients (p<0.05).

The liver injury in our cohort was suspected to be multifactorial, involving excessive cytokine release, viral infiltration of the hepatocytes, and cholangiocytes playing a role in transaminitis. The mean (±SD) duration of hospital stay was 13.5 ±15 days with 33% admissions to the ICU. The overall mortality was around 27%, with no significant difference between African Americans and Caucasians. However, patients admitted to the ICU had a very high mortality rate (54%) compared to those admitted to intermediate care (IMC)/acute care who had less severity of illness and associated pulmonary complications.

Conclusions

This study evaluates the presence of comorbidities such as DM, HTN, and obesity in patients hospitalized with COVID-19 at a community hospital in the Mid-Atlantic region of the United States. Statistical analysis of the data obtained for this cohort revealed a high frequency of GI symptoms, with diarrhea as the predominant common initial manifestation of the disease. Serum AST elevations were common and correlated with inflammatory markers and AKI. Male gender was also significantly associated with the development of AKI, higher frequency of cardiovascular events, and increased mortality. Overall mortality was noted to be 27%, with higher mortality in patients admitted to the ICU (54%) as compared to the IMC/floor (13%). These observations should spur future investigations into the role of these comorbidities, development of diarrhea, and hepatic dysfunction in COVID-19.

## Introduction

Coronavirus disease 2019 (COVID-19) is caused by a virus called the severe acute respiratory syndrome coronavirus 2 (SARS-CoV-2). This name was chosen because the virus is genetically related to the coronavirus responsible for the SARS outbreak of 2003. While related, the two viruses are different from each other in various ways [1]. The magnitude of the ongoing COVID-19 pandemic can be gathered from the rising number of cases being reported worldwide. The number of COVID-19 cases as of April 2021 is about 131 million globally with 2.85 million deaths and about 31 million cases with 0.55 million deaths in the United States [2]. Initial studies on this viral illness emerged from East Asia, which reported the lungs to be the primary target of this virus. A study by Yang et al. from Wuhan, China was one of the initial reports to suggest the extrapulmonary manifestations of the SARS-CoV-2 infection. Several recent reports on hospitalized patients with COVID-19 highlight the pulmonary, renal, and cardiac manifestations of this highly transmissible disease, which is caused by a virus that is primarily pulmonary. These extrapulmonary complications include liver dysfunction, gastrointestinal (GI) hemorrhage, acute kidney injury (AKI), and cardiac injury [3]. Early reports on GI symptoms associated with COVID-19 suggested manifestations such as diarrhea on admission in only a small percentage of the hospitalized patients (3.8-5%) [4-7]. However, a multi-center study by Qian et al. noted a higher frequency of diarrheal (21%) and other GI symptoms [anorexia (23%), nausea (11%), vomiting (6%)] [8]. A recent multicenter cohort study from Massachusetts has also noted a higher prevalence of GI symptoms (61.3%) in this group of patients [9].

In our study, we have reviewed our experience of managing COVID-19 illness at a community hospital in Baltimore, Maryland. During the early spring of 2020, a dramatic rise in the number of these cases admitted to the hospital was observed. We also observed a very high frequency of GI symptoms and hepatic dysfunction while treating hospitalized patients with COVID-19, which led to the initiation of this study. In this study, our goal was to examine the prevalence of GI manifestations and analyze the element of liver dysfunction in this population. In addition, we have also recorded inflammatory markers and other organ dysfunctions such as AKI and cardiovascular complications. Furthermore, we have also examined the outcomes, duration of hospital stay, frequency of ICU admission, and mortality in this patient population.

## Materials and methods

All hospitalized patients with a confirmed diagnosis of COVID-19 were included in the study. The diagnosis of COVID-19 infection in each patient was based on a positive polymerase chain reaction (PCR) assay with a nasopharyngeal swab. Approval by the institutional review board (IRB) for the electronic medical record (EMR) review was obtained prior to the commencement of this study. A list of COVID-19-positive hospitalized patients was obtained from the Analytics Center of Excellence at Sinai Hospital, Baltimore, and the study data were collected by reviewing patient records in the EMR system.

A total of 204 consecutive patients who were hospitalized at Sinai Hospital of Baltimore in Maryland, from March 17 to May 17, 2020, were included in this observational study. This timeframe correlates with the first two months of COVID-19-related admissions to our institution. These medical records were reviewed and the data of 184 patients were recorded and tabulated using Microsoft Excel. A total of 20 patients were excluded based on the following criteria: pediatric patients, patients not admitted to the hospital, patients who were directed to the hospice unit or Labor and Delivery unit, and patients with insufficient data for analysis. Demographics, clinical GI symptoms, and lab findings were recorded for each patient. Lab findings were analyzed for the following categories: biomarkers of inflammation [lactate dehydrogenase (LDH), ferritin, C-reactive protein (CRP)], liver enzymes [aspartate aminotransferase (AST), alanine aminotransferase (ALT), alkaline phosphatase (ALP), total bilirubin] and coagulation markers [prothrombin time (PT), international normalized ratio (INR), D-dimer, platelets]. All patient records were followed up until discharge or demise during the hospital course; the duration of hospitalization and mortality rates were also recorded.

Data collection

Data was collected from the EMR for demographics including age, sex, gender, and ethnicity. We also recorded the primary diagnosis for each patient, comorbidities, body mass index (BMI), as well as GI and hepatic manifestations of COVID-19 infection during hospitalization. The GI symptoms included in our tabulation were as follows: anosmia, dysgeusia, anorexia, nausea, emesis, dysphagia, abdominal pain, GI bleed, and diarrhea. These symptoms were self-reported by the patients. Furthermore, we also recorded the history of aspirin intake (aspirin 81 mg) prior to admission. Patients who did not provide any history regarding medications or those for whom the medication list was unavailable were excluded from the study.

Diarrhea was reported by the patients upon hospital admission or during hospitalization as loose stools and increased frequency of bowel movements per day (>3 bowel moments per day). Emesis, bloody or non-bloody, was recorded if there was at least one episode prior to or during hospitalization. Anorexia, nausea, anosmia, dysgeusia, dysphagia, and abdominal pain were all subjectively described by the admitted patients. GI bleed was clinically observed as melena, hematochezia, and/or hematemesis. Obesity was evaluated based on BMI ≥30.0 where data was available. Weight was obtained either by the standing scale or bed scale. Height was documented either by physical measurement or the patient’s stated height, for the calculation of BMI.

The first set of laboratory markers obtained from admission was tabulated, which included AST, ALT, ALP, LDH, CRP, and D-dimer. Cut-off values for laboratory markers were based on the hospital laboratory reference ranges. Values greater than the following were considered abnormal: D-dimer: ≥0.5 mg/L, AST: ≥38 U/L, ALT: ≥79 U/L in females and ≥50 U/L in males, ALP: ≥118 U/L, INR: ≥1.2, total bilirubin: ≥1.3 mg/dL, ferritin: ≥366 ng/mL in males and ≥149 ng/mL in females, CRP: ≥10 mg/L, and LDH: ≥247 U/L. Acute renal failure on admission was recorded in patients who had creatinine levels of >1 mg/dL or had presented to the hospital with a creatinine level higher than their baseline levels as documented during previous hospital visits. Hospital course and diagnostic studies for each patient were also reviewed to include any adverse GI, cardiac, vascular, and renal events.

Statistical analysis

Descriptive statistics were applied to the demographics, comorbidities, and biomarkers of interest in the study sample. Chi-square tests were used to evaluate the unadjusted associations between these patient characteristics (demographics, comorbidities, elevated biomarkers, etc.) and the outcomes of interest (AKI, cardiovascular complications, and in-hospital mortality). Multivariate logistic regression models were constructed to estimate independent associations of interest while adjusting for potential covariates. The final regression models for each outcome were adjusted for demographic factors (age, gender, race, BMI) comorbidities [hypertension (HTN), type 2 diabetes, chronic kidney disease (CKD)] and liver function tests (AST, ALT, ALP, and bilirubin) and biomarkers of inflammation (CRP, ferritin, LDH, and D-dimer). Statistical significance was defined as p<0.05. All statistical analyses were performed using the SAS software version 9.4.1 (SAS Institute, Cary, NC).

## Results

Data for this study was collected during the initial occurrence of COVID-19 cases in the Baltimore metropolitan area. A total of 184 patients diagnosed during that period with COVID-19 (via PCR nasopharyngeal tests) were included in the analysis. The demographical and clinical aspects of the study group are listed in Table 1. A majority of SARS-CoV-2-positive patients hospitalized at the Sinai Hospital during this time were African Americans (68.0%) and 51% were males. Other clinical characteristics observed included obesity in 40.6% (BMI >30) and age greater than 65 years in 60% of the cohort.

Comorbidities such as HTN, diabetes, CKD, and obesity (BMI >30) were common among most of the infected patients, with HTN being the most common condition (66.3% of patients). Obesity was also reported in a high proportion (40.6%) of hospitalized patients (Table 1). Similar studies in the past have also reported that HTN is the most common comorbidity among COVID-19 patients. In fact, a systematic review and meta-analysis of severe and fatal cases of COVID-19 showed that HTN was prevalent in 47.7% of severe cases and 47.9% of fatal cases compared to 14.3% of total cases. Diabetes was the next most prevalent condition with a 24.9% incidence among the fatal cases and 9.7% among the total cases.

**Table 1:** Demographics and clinical characteristics of 184 hospitalized COVID-19 patients between March-May 2020 COVID-19: coronavirus disease 2019; SD: standard deviation; BMI: body mass index

Characteristics	Values
Average age ≥65 years	60.0%; mean ±SD: 63.6 ±16.7
Gender (%)	
Male	51.10%
Female	48.90%
Ethnicity (%)	
African American	68.00%
Caucasian	26.00%
Hispanic	4.40%
Asian	0.55%
Unidentified	1.60%
Comorbidities	
Obesity (BMI >30 kg/m^2^), %	40.60%
Hypertension (%)	66.30%
Diabetes mellitus (%)	43.50%
Chronic kidney disease (%)	16.90%

**Figure 1:**
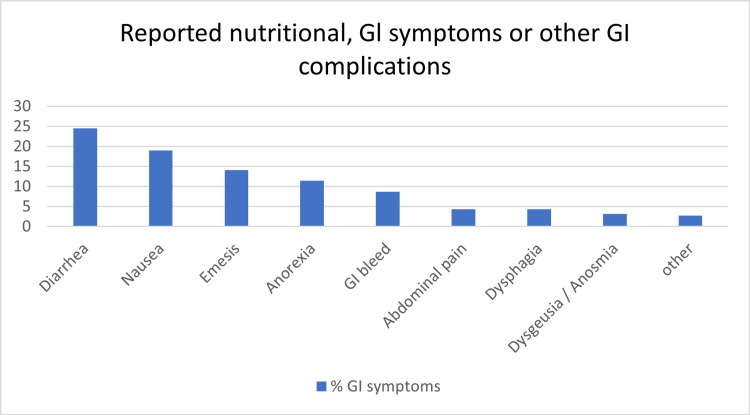
Percentage of patients reporting GI symptoms during their hospitalization GI: gastrointestinal

At least one GI manifestation was observed in 54% of the patients, of which diarrhea (24.5%) was appreciably the most common, followed by nausea (19.0%). Other prominent GI symptoms included emesis (14.1%), anorexia (11.4%), and GI bleeding (8.7%) (Figure 1).

Abdominal pain (4.3%), dysphagia (4.3%), dysgeusia and/or anosmia (3.2%) were observed in a minority of patients. It is noteworthy that out of the 16 patients (8.7%) who developed GI hemorrhage in our study group, 13 were found to be critically ill during the course of hospitalization and required intensive care. Liver test abnormalities have been frequently reported in patients with COVID-19. In our study, more than two-thirds of the patients had at least one liver test abnormality (69%). Interestingly, both ALT and ALP elevation was observed in only 25.1% and 15.9% of patients, respectively. Elevated AST was reported in 68.4% of the total patients on admission with mild AST elevations (1-2x normal) in 58.9% and moderate AST elevation (>3x normal) in 21.8% of patients.

Elevated markers of inflammation were observed in most patients with SARS-CoV-2 infection. CRP (97.7%) was elevated in almost all of the patients, followed by LDH in 86.3% and ferritin (an acute-phase reactant) in 84.9%. Both elevated AST (p-values: LDH: <0.0001, ferritin: <0.0001) and ALT (p-values: LDH: <0.0001, ferritin: <0.0115) correlated with high levels of LDH and ferritin. Interestingly, CRP was not linked with elevated transaminases in our study.

AKI correlated with elevated AST (p<0.0097), increased total bilirubin (p<0.017), and high INR values (p<0.012). Coagulation factors measured, owing to the prothrombotic effects of the SARS-CoV-2, included D-dimers and PT. Not surprisingly, D-dimer was elevated in 89.9% of the patients and abnormal PT was observed in 98.2%. In addition, elevated D-dimers and PT also showed a positive correlation with elevated AST numbers on the Pearson correlation coefficient (p<0.0001). Moreover, elevated AST and ALT levels correlated with AKI as well (Table 2).

**Table 2:** Association of liver function abnormality and markers of inflammation with AKI, cardiovascular events, and mortality AKI: acute kidney injury; AST: aspartate aminotransferase; ALT: alanine aminotransferase; ALP: alkaline phosphatase; CRP: C-reactive protein; LDH: lactate dehydrogenase; INR: international normalized ratio

Variables	Mortality, %	P-value	AKI, %	P-value	Cardiovascular events, %	P-value
AST						
Normal	16.40%	0.23	21.80%	0.029	20.00%	0.96
Elevated	29.40%		42.00%		14.30%	
ALT						
Normal	25.40%	0.17	32.80%	0.12	16.40%	0.85
Elevated	26.70%		42.40%		11.10%	
ALP						
Normal	25.50%	0.53	35.30%	0.78	17.00%	0.16
Elevated	31.00%		34.50%		10.30%	
Total bilirubin						
Normal	26.50%	0.21	32.50%	0.045	15.70%	0.5
Elevated	21.40%		64.30%		21.40%	
CRP						
Normal	25.00%	0.96	25.00%	0.96	25.00%	0.96
Elevated	26.20%		35.10%		15.50%	
Ferritin						
Normal	28.00%	0.98	20.00%	0.041	12.00%	0.22
Elevated	28.40%		40.40%		17.00%	
LDH						
Normal	16.70%	0.19	20.80%	0.39	16.70%	0.43
Elevated	28.50%		37.80%		15.90%	
D-dimer						
Normal	12.50%	0.65	6.25%	0.59	6.25%	0.69
Elevated	27.90%		40.60%		15.40%	
INR						
Normal	22.50%	0.43	28.30%	0.098	16.70%	0.62
Elevated	34.40%		46.90%		15.60%	

**Figure 2:**
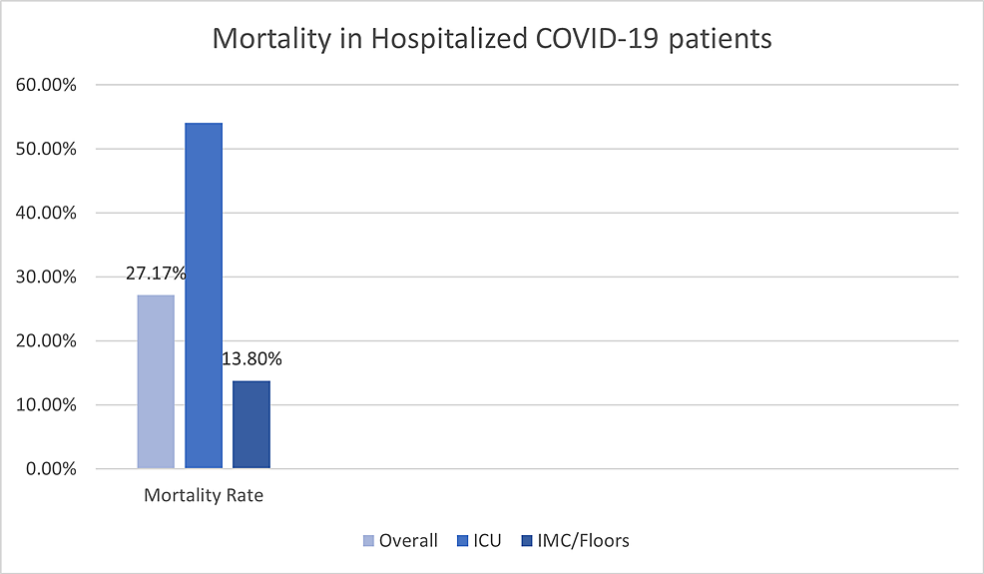
Mortality in hospitalized COVID-19 patients COVID-19: coronavirus disease 2019; ICU: intensive care unit; IMC: intermediate care unit

The mean (±SD) duration of hospital stay was 13.51 (±15.34) days (median: nine days; range: 2-164 days). The overall mortality in our group of patients was 27.17%. The mortality rate in African American patients was 27% as compared to a mortality rate of 25% in the Caucasian population. The difference in mortality among ethnic groups was not statistically significant. Furthermore, the mortality in this group of patients in the ICU setting was found to be higher (54.09%) than mortality in patients admitted to intermediate care (IMC)/floors (13.8%) (Figure 2).

Male gender was significantly associated with the development of AKI (p<0.0042), cardiovascular events (<0.021), and increased mortality (p<0.033) (Table 3). Adverse outcomes in other studies have been predicted based on the trends in inflammatory markers. Interestingly, AST was found to have a statistically significant relationship with mortality on bivariate analysis and also approached significance in the multivariate regression model. Other factors such as male gender (p<0.04) and advanced age (p<0.01) also demonstrated an increased association with mortality.

**Table 3:** Correlation of demographics and comorbidities with complications including mortality, AKI, and cardiovascular events AKI: acute kidney injury; BMI: body mass index; HTN: hypertension; DM: diabetes mellitus; CKD: chronic kidney disease

	Mortality p-value	AKI p-value	Cardiovascular events p-value
Age	0.013	0.049	0.6
Male gender	0.033	0.0042	0.021
BMI	0.85	0.082	0.11
HTN	0.45	0.0055	0.79
DM	0.63	0.27	0.58
CKD	0.43	0.31	0.91

There were 56 hospitalized patients (31.6%) who had been taking aspirin at home prior to their admission to the hospital. The mortality rate in this group of 56 patients was 32.1% as compared to the 68% mortality rate in patients who were not on aspirin. The difference in mortality rate between these two groups was not significant (p<0.3).

## Discussion

This observational study illustrates the findings among the SARS-CoV-2-positive patient population admitted to a community hospital in the Mid-Atlantic region. It is important to note that these observations were interpreted in the setting of limited diagnostic and therapeutic modalities during the early stages of the ongoing COVID-19 pandemic. The salient findings have been compared with observations reported from China, Europe, and other parts of the United States. All 184 consecutive patients who tested positive for SARS-CoV-2 on a nasopharyngeal swab test and subsequently hospitalized were included in our study. Although it is well recognized that the SARS-CoV-2 virus is primarily a pulmonary virus and most of the infected patients present with fever and upper respiratory symptoms, a significant number of patients reported GI symptoms at the time of admission. This was in contrast with earlier reports from China, which revealed a very low prevalence (<5%) of patients with GI symptomatology [10-15].

Gastrointestinal symptoms

We observed that 53.8% of our cohort had GI symptoms, most commonly diarrhea, nausea, and anorexia (Figure 1). Similar observations were reported in a study by Elmunzer et al., in which 53% of patients with COVID-19 infection demonstrated at least one GI symptom on admission to various area hospitals in North America [16]. A study from Louisiana by Price-Haywood et al. compared manifestations of COVID-19 between White and African American populations and showed that 14-49% of patients admitted had abdominal pain and diarrhea [17]. Also, the frequency of GI symptoms was quite high (61.3%) in patients admitted to a hospital in Massachusetts as reported by Redd et al. [9]. A large meta-analysis of data from Hong Kong reported that 17.6% of patients with COVID-19 positivity had GI symptoms during hospital admission, which is lower compared to our findings [18].

Such distinct variations in the numerical data related to GI symptomatology from different parts of the world suggest varying phenotypic expression of the disease or different strains of the same virus. In addition, it may also be related to variability in data collection, where the patients may not have been asked questions related to the digestive system.

Diarrhea, reported either prior to hospital admission or during hospitalization, was present in 24.5% of our patients, followed by nausea as the second most common symptom. It should be noted that the definition of diarrhea was subjective as the details of the diarrheal illness were not recorded or tabulated. Similar observations have been reported independently by investigators in different parts of the world including Europe and Canada [19-25]. Lechien et al. reported an incidence of diarrhea in 38.1% of patients admitted at 18 European hospitals (N=1,420) with SARS-CoV-2. Similarly, in a study from Canada, O'Brien et al. reported that 34-44% of hospitalized patients had frequent loose stools [26-27]. Other studies conducted in the United States have also shown an increased frequency of GI symptoms and diarrhea [16], whereas a meta-analysis of the Chinese population indicated that diarrhea was found in as low as 12.5% of the pooled cohort [18].

Although the underlying reason for diarrhea in this population remains unclear, the use of antibiotics and other drugs before or after hospitalization may have contributed to this higher frequency. It has been reported that viral RNA is excreted in the feces of patients who have the infection and has also been detected in the GI epithelial cells via mucosal biopsies of the GI tract. The presence of the virus in the intestinal epithelial cells may imply direct effects of the virus on the absorptive process of the small intestine in this group of patients. In a recent study by Jiao et al., five rhesus macaques were intranasally inoculated with SARS-CoV-2, leading to infection and pathologic changes in respiratory as well as in digestive tissues. Furthermore, in this study, expectedly, intragastric inoculation with SARS-CoV-2 also resulted in the infection of digestive tissues and inflammation in both the lung and digestive tissues. In fact, the persistence of viral RNA in the stool samples even after a negative nasopharyngeal test would suggest that detection of COVID-19 virus in the stool may be a longer-lasting marker of infectivity in these patients [18,28].

Less commonly reported GI manifestations that were observed in our study included emesis, GI hemorrhage, dysgeusia, and/or anosmia (Figure 1). Dysgeusia and anosmia were perhaps underreported in the data as these symptoms are not often inquired about or necessarily reported by patients. In fact, although these symptoms are clinically frequent, they have rarely been reported in other studies in the US. Minor GI bleed was noted in 8.7% (n=16) of patients, of which nine patients experienced only upper GI bleed while three patients had only lower GI bleed and four patients had both upper and lower GI bleed. It is noteworthy that endoscopic intervention was required in three of the 13 critically ill patients with GI hemorrhage who were admitted to the ICU.

Prevalence of transaminitis in COVID-19 patients

In our study, abnormal liver tests indicating acute liver injury were observed in 69% of the total study population. Comparable observations were documented on the first encounter in a larger study by Hundt et al. where 66.9% had abnormal liver tests [29]. In a study from Massachusetts, Bloom et al. have reported elevated liver tests on admission in 69% of patients, which is similar to our findings [10]. In contrast, cross-sectional studies from China have reported abnormal liver tests in <50% of the study populations on admission [30].

Among liver tests, serum AST was found to be elevated in 68.4% of all COVID-19-positive patients while serum ALT elevation was noted to be much lower (25.1%). Mild elevations (1-2x ULN) in serum AST were found in 58.9% of patients, and moderate elevation (>3x ULN) in 21.8% of patients on admission. Increased AST levels were also reported by Hundt et al. (1-2x ULN; AST: 63.7%) [29]. In addition, similar observations were reported in retrospective studies conducted in New York, which showed AST elevations in 49-57% of COVID-19 patients. The study from Louisiana also reported a rise in AST between 55-62% in their study cohort [31-33].

Clinically significant mild to moderate elevations in AST and ALT are not described so far; however, they may suggest acute hepatic injury from the SARS-CoV-2 virus. The presence of the virus in liver cells may lead to cellular dysfunction due to the systemic inflammatory response triggered by the binding of the virus to angiotensin-converting enzyme 2 (ACE2) on cholangiocytes, thereby causing liver injury [34]. AST may also arise from extrahepatic sources such as myocardial cells, lung, kidney, and red blood cells [35] and may be related to inflammatory markers, with which it correlated significantly in our study. These observations may warrant further examination of AST release due to viral liver injury [16,35].

Liver enzymes in relation to inflammatory markers and mortality

A positive correlation was observed between AST elevations and the levels of inflammatory markers in blood such as ferritin (p<0.0001) and LDH (p<0.0001). We postulate that a positive correlation between elevated AST and inflammatory markers may have occurred as a result of the SARS-CoV-2 virus-induced inflammation leading to acute liver injury. It is noteworthy that the autopsy studies in patients who died of SARS have also shown the presence of viral particles in the liver tissue [36]. More recently, the presence of viral particles was detected in the liver tissue in rhesus macaques intranasally inoculated with SARS-CoV-2 [37]. Furthermore, the affinity of the virus for ACE2 protein receptors on cholangiocytes and hepatocytes may cause hepatobiliary injury [38]. This has been observed in other studies that have measured increased levels of gamma-glutamyl-transferase (GGT) in patients with the COVID-19 virus, suggesting injury to cholangiocytes by the virus [36].

In our study, serum AST was also noted to be high on admission in a large number of patients who subsequently expired (71.4%). And there was a statistical correlation between AST and mortality on bivariate analysis (p=0.229), presumably due to the fact that AST elevation indicates increased systemic inflammatory response resulting in multi-organ injury in this group of patients. However, when analyzed based on the multivariate regression model, the association approached but did not reach statistical significance. Clearly, this observation suggests that liver abnormalities may indicate a more severe COVID-19 infection, as reported in other studies where the persistent elevation of liver enzymes has been associated with a more severe outcome. Perhaps, further studies with a larger sample size may better illustrate the relationship between AST and mortality based on AST/ALT levels on admission.

The use of low-dose aspirin to prevent thrombosis and cardiovascular disease is extensively described in the literature. Our results showed that the use of low-dose aspirin intake was not associated with any significant reduction in mortality. A similar study has also reported no reduction in mortality while on low-dose aspirin [39]. Furthermore, a recent meta-analysis by Salah et al. supported these findings as no association between the use of aspirin and mortality in patients with COVID-19 was observed. It is noteworthy that the patients on aspirin were older, and had preexisting coronary artery disease or diabetes mellitus (DM) [40].

Acute kidney injury related to liver enzymes in SARS-CoV-2 infection

In this study, AKI as defined by our criteria was found in 34.8% of the patients hospitalized with COVID-19. It is notable that there was a strong correlation between elevated AST and AKI, and for most of the patients who had elevated AST levels on admission, AKI was present in a statistically significant number (42.0%, p<0.028). These observations suggest a common factor between the two variables, which we believe is the systemic inflammatory response. Furthermore, AKI also correlated significantly with the male gender (p<0.005) and patients who had known HTN (p<0.01). Interestingly, AKI also correlated with elevated bilirubin (p<0.05) and elevated ferritin (p<0.05). Similar observations have been reported in a study on the urban population from New Orleans, which reported digestive symptoms in relation to acute renal insufficiency [41].

Duration of hospital stay and outcomes in hospitalized COVID-19 patients

In our study, the mean (±SD) duration of hospital stay was 13.51 (±15.34) days (median: nine days; range: 2-164 days). These observations are similar to the outcomes reported by Mallow et al.; in their study, the mean hospital stay was noted to be 8.9 days, while it was 7.6 days in the ICU setting [42]. It is noteworthy that in our study, the mortality was much higher in the ICU setting (54.09%) as compared to the patients admitted to IMC/floors (13.8%); the overall mortality was noted to be 27.17%. Similar observations have been reported in previous studies from Louisiana and Cincinnati with mortality rates ranging between 22-30%. Furthermore, in our study, the analysis of outcomes in hospitalized patients with COVID-19 infection suggested that a majority of the patients (22 from ICU and 97 from floors) were discharged home, while a small number (four from ICU and nine from floors) were moved to hospice care.

Limitations

This study has all the drawbacks that are commonly associated with a retrospective analysis; however, we believe we have put acute findings related to COVID-19 into perspective, which may allow readers and researchers to focus on select areas in relation to COVID-19 illness such as liver dysfunction, diarrhea, and their association with clinical outcomes such as AKI and/or severity of the viral disease.

## Conclusions

This study analyzes the presence of comorbidities such as DM, HTN, and obesity in patients hospitalized with COVID-19 illness. Close examination of these observations and statistical analysis of the data in this cohort of patients diagnosed in the early part of the pandemic suggest that diarrhea is a common initial manifestation of the disease. Serum AST elevations are indicative of inflammatory response to the virus through cytokine storm; the stronger correlation between AST and AKI and associated mortality indicate inflammatory response to the virus as the likely explanation for GI symptoms and hepatic dysfunction in these patients. Male gender was significantly associated with the development of AKI (p<0.005), cardiovascular events (p<0.05), and increased mortality (p<0.05). Overall mortality was noted to be 27%, with ICU-related mortality being higher (54%) compared to those in the IMC/floors (13%). Future studies should investigate the role of these comorbidities, cytokine storms, and cellular damage directly caused by the coronavirus in the development of GI manifestations and liver dysfunction.

## References

[1] (2022). WHO: novel coronavirus 2019. https://www.who.int/emergencies/diseases/novel-coronavirus-2019/technical-guidance/naming-the-coronavirus-disease--and-the-virus-that-causes-it.

[2] (2022). Johns Hopkins Medicine: coronavirus map. https://coronavirus.jhu.edu/map.html.

[3] Xiang YT, Li W, Zhang Q (2020). Timely research papers about COVID-19 in China. Lancet.

[4] Yang X, Yu Y, Xu J (2020). Clinical course and outcomes of critically ill patients with SARS-CoV-2 pneumonia in Wuhan, China: a single-centered, retrospective, observational study. Lancet Respir Med.

[5] Guan WJ, Ni ZY, Hu Y (2020). Clinical characteristics of coronavirus disease 2019 in China. N Engl J Med.

[6] Huang C, Wang Y, Li X (2020). Clinical features of patients infected with 2019 novel coronavirus in Wuhan, China. Lancet.

[7] Zhou F, Yu T, Du R (2020). Clinical course and risk factors for mortality of adult inpatients with COVID-19 in Wuhan, China: a retrospective cohort study. Lancet.

[8] Qian GQ, Yang NB, Ding F (2020). Epidemiologic and clinical characteristics of 91 hospitalized patients with COVID-19 in Zhejiang, China: a retrospective, multi-centre case series. QJM.

[9] Redd WD, Zhou JC, Hathorn KE (2020). Prevalence and characteristics of gastrointestinal symptoms in patients with severe acute respiratory syndrome coronavirus 2 infection in the United States: a multicenter cohort study. Gastroenterology.

[10] Bloom PP, Meyerowitz EA, Reinus Z (2021). Liver biochemistries in hospitalized patients with COVID-19. Hepatology.

[11] Zhou Z, Zhao N, Shu Y, Han S, Chen B, Shu X (2020). Effect of gastrointestinal symptoms in patients with COVID-19. Gastroenterology.

[12] Jin X, Lian JS, Hu JH (2020). Epidemiological, clinical and virological characteristics of 74 cases of coronavirus-infected disease 2019 (COVID-19) with gastrointestinal symptoms. Gut.

[13] Zhang JJ, Dong X, Cao YY (2020). Clinical characteristics of 140 patients infected with SARS-CoV-2 in Wuhan, China. Allergy.

[14] Han C, Duan C, Zhang S (2020). Digestive symptoms in COVID-19 patients with mild disease severity: clinical presentation, stool viral RNA testing, and outcomes. Am J Gastroenterol.

[15] Du M, Cai G, Chen F, Christiani DC, Zhang Z, Wang M (2020). Multiomics evaluation of gastrointestinal and other clinical characteristics of COVID-19. Gastroenterology.

[16] Elmunzer BJ, Spitzer RL, Foster LD (2021). Digestive manifestations in patients hospitalized with coronavirus disease 2019. Clin Gastroenterol Hepatol.

[17] Price-Haywood EG, Burton J, Fort D, Seoane L (2020). Hospitalization and mortality among black patients and white patients with Covid-19. N Engl J Med.

[18] Cheung KS, Hung IF, Chan PP (2020). Gastrointestinal manifestations of SARS-CoV-2 infection and virus load in fecal samples from a Hong Kong cohort: systematic review and meta-analysis. Gastroenterology.

[19] Mao R, Qiu Y, He JS (2020). Manifestations and prognosis of gastrointestinal and liver involvement in patients with COVID-19: a systematic review and meta-analysis. Lancet Gastroenterol Hepatol.

[20] Cholankeril G, Podboy A, Aivaliotis VI, Pham EA, Spencer SP, Kim D, Ahmed A (2020). Association of digestive symptoms and hospitalization in patients with SARS-CoV-2 infection. Am J Gastroenterol.

[21] Wu J, Liu J, Zhao X (2020). Clinical characteristics of imported cases of coronavirus disease 2019 (COVID-19) in Jiangsu Province: a multicenter descriptive study. Clin Infect Dis.

[22] Liu K, Fang YY, Deng Y (2020). Clinical characteristics of novel coronavirus cases in tertiary hospitals in Hubei Province. Chin Med J (Engl).

[23] Luo S, Zhang X, Xu H (2020). Don't overlook digestive symptoms in patients with 2019 novel coronavirus disease (COVID-19). Clin Gastroenterol Hepatol.

[24] Yang W, Cao Q, Qin L (2020). Clinical characteristics and imaging manifestations of the 2019 novel coronavirus disease (COVID-19): a multi-center study in Wenzhou city, Zhejiang, China. J Infect.

[25] Xu XW, Wu XX, Jiang XG (2020). Clinical findings in a group of patients infected with the 2019 novel coronavirus (SARS-Cov-2) outside of Wuhan, China: retrospective case series. BMJ.

[26] O'Brien J, Du KY, Peng C (2020). Incidence, clinical features, and outcomes of COVID-19 in Canada: impact of sex and age. J Ovarian Res.

[27] Lechien JR, Chiesa-Estomba CM, Place S (2020). Clinical and epidemiological characteristics of 1420 European patients with mild-to-moderate coronavirus disease 2019. J Intern Med.

[28] Wu Y, Guo C, Tang L (2020). Prolonged presence of SARS-CoV-2 viral RNA in faecal samples. Lancet Gastroenterol Hepatol.

[29] Hundt MA, Deng Y, Ciarleglio MM, Nathanson MH, Lim JK (2020). Abnormal liver tests in COVID-19: a retrospective observational cohort study of 1,827 patients in a major U.S. hospital network. Hepatology.

[30] Cai Q, Huang D, Yu H (2020). COVID-19: abnormal liver function tests. J Hepatol.

[31] Sultan S, Altayar O, Siddique SM (2020). AGA Institute rapid review of the gastrointestinal and liver manifestations of COVID-19, meta-analysis of international data, and recommendations for the consultative management of patients with COVID-19. Gastroenterology.

[32] Ferm S, Fisher C, Pakala T (2020). Analysis of gastrointestinal and hepatic manifestations of SARS-CoV-2 infection in 892 patients in Queens, NY. Clin Gastroenterol Hepatol.

[33] Phipps MM, Barraza LH, LaSota ED (2020). Acute liver injury in COVID-19: prevalence and association with clinical outcomes in a large U.S. cohort. Hepatology.

[34] Chai X, Hu L, Zhang Y (2020). Specific ACE2 expression in cholangiocytes may cause liver damage after 2019-nCoV infection [PREPRINT]. bioRxiv.

[35] Vroon DH, Israili Z (1990). Aminotransferases. Clinical Methods, the History, Physical, and Laboratory Examinations. 3rd Edition.

[36] Farcas GA, Poutanen SM, Mazzulli T (2005). Fatal severe acute respiratory syndrome is associated with multiorgan involvement by coronavirus. J Infect Dis.

[37] Jiao L, Li H, Xu J (2021). The gastrointestinal tract is an alternative route for SARS-CoV-2 infection in a nonhuman primate model. Gastroenterology.

[38] Hamming I, Timens W, Bulthuis ML, Lely AT, Navis G, van Goor H (2004). Tissue distribution of ACE2 protein, the functional receptor for SARS coronavirus. A first step in understanding SARS pathogenesis. J Pathol.

[39] Yuan S, Chen P, Li H, Chen C, Wang F, Wang DW (2021). Mortality and pre-hospitalization use of low-dose aspirin in COVID-19 patients with coronary artery disease. J Cell Mol Med.

[40] Salah HM, Mehta JL (2021). Meta-analysis of the effect of aspirin on mortality in COVID-19. Am J Cardiol.

[41] Mohamed MB, Lukitsch I, Torres-Ortiz AE (2020). Acute kidney injury associated with coronavirus disease 2019 in urban New Orleans. Kidney360.

[42] Mallow PJ, Belk KW, Topmiller M, Hooker EA (2020). Outcomes of hospitalized COVID-19 patients by risk factors: results from a United States hospital claims database. J Health Econ Outcomes Res.

